# Photodynamic Stromal Depletion in Pancreatic Ductal Adenocarcinoma

**DOI:** 10.3390/cancers15164135

**Published:** 2023-08-16

**Authors:** Nicole Lintern, Andrew M. Smith, David G. Jayne, Yazan S. Khaled

**Affiliations:** 1 School of Biomedical Sciences, University of Leeds, Leeds LS2 9JT, UK; 2 Leeds Institute of Medical Research, St James’s University Hospital, Leeds LS9 7TF, UK

**Keywords:** pancreatic ductal adenocarcinoma, photodynamic therapy, stroma, tumour microenvironment, extracellular matrix, fibrosis, cancer therapeutics

## Abstract

**Simple Summary:**

Pancreatic cancer remains one of the deadliest cancers and has a dismal survival rate. The disease is known to be resistant to chemo- and radiotherapy, and surgery can only be curative in a small percentage of patients. It is thought that the thick tissue (stroma) surrounding the cancer cells is the main factor in the resistance to systemic therapy. Photodynamic therapy (PDT) is a new treatment that uses a specific light to activate a smart molecule to cause cancer cell death. Emerging evidence suggests that PDT may offer a solution that can alter the stroma and kill pancreatic cancer cells. The aim of this review is to summarise the literature and explore the effect of PDT on the stroma within pancreatic cancer in order to improve our understanding of this new therapy and its application.

**Abstract:**

Pancreatic ductal adenocarcinoma (PDAC) is one of the deadliest solid malignancies, with a five-year survival of less than 10%. The resistance of the disease and the associated lack of therapeutic response is attributed primarily to its dense, fibrotic stroma, which acts as a barrier to drug perfusion and permits tumour survival and invasion. As clinical trials of chemotherapy (CT), radiotherapy (RT), and targeted agents have not been successful, improving the survival rate in unresectable PDAC remains an urgent clinical need. Photodynamic stromal depletion (PSD) is a recent approach that uses visible or near-infrared light to destroy the desmoplastic tissue. Preclinical evidence suggests this can resensitise tumour cells to subsequent therapies whilst averting the tumorigenic effects of tumour–stromal cell interactions. So far, the pre-clinical studies have suggested that PDT can successfully mediate the destruction of various stromal elements without increasing the aggressiveness of the tumour. However, the complexity of this interplay, including the combined tumour promoting and suppressing effects, poses unknowns for the clinical application of photodynamic stromal depletion in PDAC.

## 1. Introduction

Pancreatic cancer was responsible for 466,000 deaths worldwide in 2020; yet, the total number of cases diagnosed was only marginally greater (496,000) [1]. It is the seventh leading cause of cancer death across the sexes [1], though projections show it will rise to third place by 2025 [2]. This is in part due to the lack of biomarkers and imaging tools for early detection; it is also due to its aggressiveness [3]. Pancreatic ductal adenocarcinoma (PDAC) accounts for 85–95% of all solid pancreatic tumours [4] and usually originates in the exocrine cells of the pancreatic ducts. One of the most distinct features of PDAC is its high stromal content, which can constitute 90% of the tumour [5]. The complex interplay between stromal components and cancer cells permits both disease initiation and progression, including metastasis to other organs [6,7]. The greatest challenge in PDAC treatment is the continued production and deposition of a fibrous extracellular matrix (ECM), which is refractory to most interventions including chemo- and immunotherapies [8]. The dense tissue gives rise to a hypovascular tumour with elevated interstitial fluid pressure (IFP), resulting in difficulties in drug delivery and distribution [9]. Because of this, resistance to current therapies remains a major challenge.

**Figure 1:**
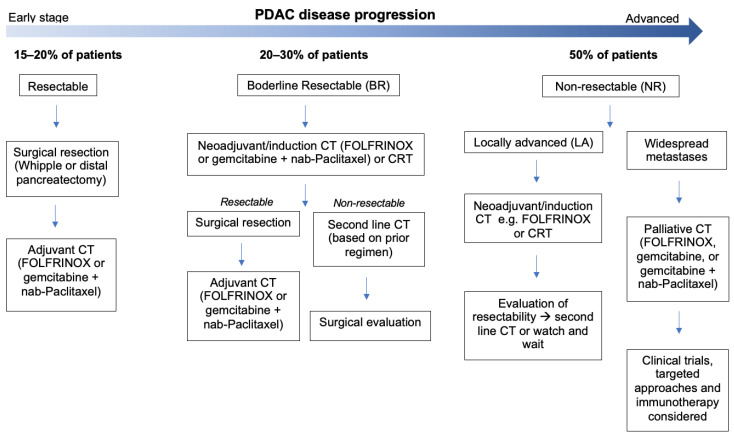
An outline of the standard treatment pathways for pancreatic ductal adenocarcinoma. At initial presentation, just 15–20% of patients are suitable for upfront resection. Adjuvant CT (AC) is the standard of care (SOC) following resection in PDAC. A greater proportion of patients present with borderline resectable (BR) disease (20–30%) and typically receive neoadjuvant CT (NACT) before surgical evaluation. Half of patients present with initially non-resectable (NR) disease, though if locally advanced (LA) surgical evaluation will be considered following NACT. As there is little evidence supporting surgical resection for metastatic PDAC, palliative CT is adopted. CT: chemotherapy.

Given that surgical resection with radical intent remains the only curative approach in the clinical management of pancreatic cancer [10], the state of disease at diagnosis is classified as resectable, borderline resectable (BR), or non-resectable (locally advanced or metastatic) according to strict criteria [11,12,13]. Treatment commences accordingly (Figure 1).

A pancreaticoduodenectomy (Whipple) or distal pancreatectomy is the most commonly performed first-line treatment for PDAC [14]. Whilst these surgeries can increase long-term survival, they infrequently achieve R0 resection and negatively impact on quality of life. Without any additional therapies, 60–80% of patients relapse within the first year [15,16,17]. Combined with the fact that just 10–20% of patients are suitable for resection at diagnosis [18], most cases require a multi-modal treatment strategy. Surgical resection followed by adjuvant CT is the standard approach for resectable PDAC following years of clinical trials suggesting its benefit to overall and disease-free survival (DFS) [19,20,21]. The standardised regimens include gemcitabine (Gem), a nucleoside analogue, or FOLFIRINOX (FFX), a combination of leucovorin, fluorouracil, irinotecan, and oxaliplatin. The latter is more effective, with one study showing a 3-year DFS of 39.7% vs. 21.4% with Gem [21]. However, 30–40% of patients fail to receive adjuvant chemotherapy due to postoperative complications [22,23,24] or early disease progression [25,26]. Of those that do begin postoperative chemotherapy, many fail to complete the course due to toxicity and decrease in performance status [27,28]. Ultimately, 60–75% of patients receiving FFX and other chemotherapies soon relapse [29,30,31,32], with the median overall survival (OS) following surgical resection and CT being ~20–40 months [33,34,35].

Due to the inadequacy of this approach, neoadjuvant chemotherapy (NAT) has recently been implemented for PDAC, mostly in the BR and locally advanced (LA) settings [36]. NAT can downstage advanced tumours to a resectable state, select patients with favourable tumour biology for surgery, improve margins, decrease node positivity, and control micrometastases [37,38,39]. A significant survival benefit has been observed with NAT for BR, LA, and early (upfront resectable) PDAC [40,41,42]. For many, the combination of adjuvant and neoadjuvant CT/RT has the greatest impact on OS [43,44,45,46,47], though treatment success can be influenced by many variables, including patient age or the location of disease in the pancreas [48,49,50,51]. Unfortunately, as ~50% of cases are metastatic at presentation, and many additional patients develop local recurrences or metastases during treatment [52,53,54], most patients end up receiving palliative CT. In this setting, one study found that OS was 11.1 months for FFX and 6.8 months for Gem, with median response rates of 31.6% and 9.4%, respectively [55]. Another study found that the response rate of metastatic PDAC to Gem was just 7%, with an OS of 6.7 months, whereas the combination of Gem plus nab-paclitaxel (GnP) gave rise to a better response of 23%, with an OS of 8.5 months [56]. FFX and GnP remain the recommended palliative treatments for PDAC [57].

As most cases of PDAC are highly refractory to systemic treatments, including chemo- and radiotherapies, and their benefit to survival is very low, various targeted agents have been investigated in combination alongside classical cytotoxics [58,59,60]. These are designed around several key areas, including PDAC cell metabolism, deoxyribonucleic acid (DNA) repair, immune response, and the tumour microenvironment (TME). Whilst some have shown promising results in early studies, few have achieved more than a marginal benefit in DFS or OS in larger randomised trials [61,62,63,64]. This lack of efficacy may stem from inter- and intra-tumoural heterogeneity, lack of specific biomarkers to predict effectiveness of target blocking, the use of specific CT regimens, adverse effects necessitating dose reduction, development of resistance, or, most notably, the dense, hypovascularised stroma [9,65,66]. The PDAC stroma contributes to drug resistance by preventing the entry of therapeutic agents into tumour cells, restricting their anti-tumour effects [67,68,69]. Destroying the stroma could alleviate this and improve patient outcomes. However, many initial anti-stromal therapies for PDAC have had toxic effects, lacked efficacy, and added little to DFS or OS [70,71,72]. Some have even been associated with accelerated PDAC progression [73,74]. An alternative solution is the use of PDT, which has not only proven to be feasible and safe for the destruction of PDAC cells [75,76,77] but might also play a role in stromal depletion [78,79,80]. Generally, PDT is well tolerated and does not depend on any specific molecular aberrations, making it applicable to more patients. One possibility is that by destroying the stroma, PDT could enhance the activity of targeted agents as well as traditional cytotoxics. This review focuses on the preclinical and clinical evidence for PDT in PDAC, with particular emphasis on the status of its anti-stromal activity. It also assesses how we must be mindful of ‘avoiding the extremes’ in designing and implementing PDT for stromal depletion in PDAC, in order to avoid causing unnecessary destruction of any tumour-suppressing properties.

## 2. The PDAC Tumour Microenvironment and Its Regulation

The PDAC tumour microenvironment (TME) describes the confinement of a pancreatic tumour and the extensive constituents that surround and support it. The TME has two compartments: the stroma and the ECM. Whilst the ECM more specifically comprises non-cellular structural proteins, including collagens I, III, IV and V, integrins, proteoglycans, and glycoproteins [81], the stroma contains an extensive mix of fibroblasts, bone marrow-derived stem cells, pancreatic stellate cells (PSCs), immune cells, neurons, and blood and lymphatic vessels, as well as the ECM [82]. The most prominent immune cells are myeloid-derived suppressor cells (MDSCs), macrophages, mast, and effector T-cells [83]. Together, these produce an immunosuppressive effect resulting in the reduction in the anti-tumour activity of the immune system [84].

In PDAC, the desmoplastic reaction describes the initial and continued production of ECM in response to an invasive carcinoma [85]. As the reaction proceeds, the area becomes stiffened and increasingly dense, and eventually, the TME adopts very different mechanical and biophysical traits compared to those of healthy pancreatic tissue [86].

**Figure 2:**
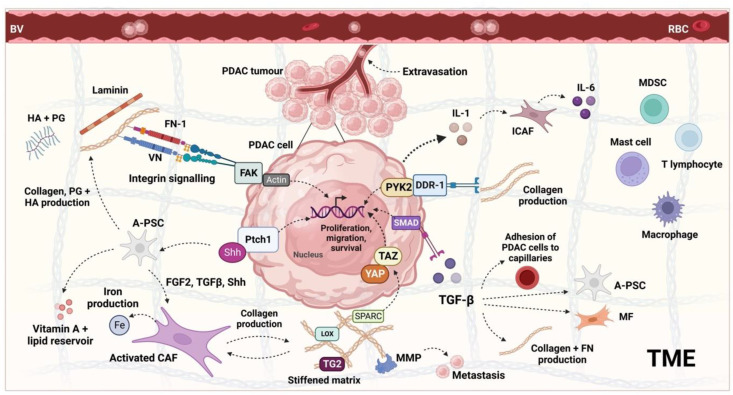
The main PDAC TME components and tumourigenic signalling pathways. The TME is a complex mixture of structural (ECM) and cellular components that play a reciprocal role in the progression of the stroma and the cancer itself. Facilitating many of the interactions between the tumour and stromal components are integrins. Via connecting proteins FN-1/VN-1, collagens interact with the integrins on the PDAC cell surface, which activates FAK and converts actin polymerisation into traction force, driving tumour cell invasion. Collagen also interacts with DDR-1, which binds and activates several signalling proteins, including PYK2, leading to the upregulation of pro-survival genes such as *Hes1* and migratory pathways via p130Cas/JNK. The result is the upregulation of N-cad, which promotes the EMT and acquisition of a motile phenotype. Upon Shh pathway activation, PSCs form CAFs, which are the greatest producer of the ECM components, including collagen, PG, and HA. Secreted proteins SPARC, LOX, and TG2 mediate collagen crosslinking, which leads to tissue stiffness, the production of further collagen, and the activation of YAP/TAZ, which promotes cell proliferation. MMPs, which are upregulated by integrin signalling, also modulate the proteolytic activity of the matrices, leading to the release of individual PDAC cells from the TME. Cytokines such as TGF-β and IL-1 released from the PDAC secretome have many functions, including promotion of further collagen production, activation of CAFs, and the adhesion of PDAC cells to capillaries. Activated fibroblasts have several functions: MFs are crucial for the continued deposition of ECM components, whereas ICAFs release various tumour-promoting inflammatory molecules such as IL-6, which enhance the formation of the immunosuppressive environment by regulating immune cell activity. They also support the metabolism of PDAC cells, such as by providing them with labile iron for survival or acting as a cellular reservoir for vitamin A and lipids. BV, blood vessel; RBC, red blood cell; PSC, pancreatic stellate cell; CAF, cancer-associated fibroblast; MDSC, myeloid-derived suppressor cell; TGF-β, transforming growth factor beta; HA, hyaluronan; FN-1, fibronectin 1; VN, vitronectin; DDR-1/2, discoidin receptor ½; PYK2, FAK-related protein tyrosine kinase; FAK, focal adhesion kinase; MMP, matrix metalloproteinase; TG2, tissue transglutimase; LOX, lysyl oxidase; Shh, sonic hedgehog; FGF2, fibroblast growth factor 2; YAP, yes-associated protein; TAZ, transcriptional co-activator with PDZ-binding motif; ICAF, inflammatory cancer-associated fibroblast; MF, myofibroblast; A-PSC, activated pancreatic stellate cell; PG, proteoglycan; SPARC, secreted protein acidic and rich in cysteine; EMT, epithelial to mesenchymal transition; IL-6, interleukin 6; IL-1, interleukin 1; Fe, iron; PDAC, pancreatic ductal adenocarcinoma; JNK, c-Jun N-terminal kinase; N-cad, n-cadherin; TME, tumour microenvironment; ECM, extracellular matrix; SMAD, mothers against decapentaplegic; Ptch1, patched-1 receptor. The figure was created on Biorender.com (accessed on 5 January 2023).

The close link between these TME components and PDAC progression (Figure 2) has led to the development of various stromal depletion strategies, though most have failed. It is theorised that this is because the TME is highly heterogeneous between and within PDAC tumours [87]. By reviewing 143 treatment-naïve resections and pre-treatment biopsies from over 200 cases of advanced PDAC, a 2021 study noted several recurrent histological patterns in the TME and named these ‘subTMEs’ [88]. There were three regions identified: ‘deserted’, characterised by a keloid or myxoid type ECM with thin, spindle-shaped fibroblasts; ‘reactive’, where the ECM comprises very few acellular components, and yet the fibroblasts are enlarged; or there are those with features of both. Comparing the TME and tumour cell samples, there were 5050 differentially expressed genes (DEGs) and 12,704 ribonucleic acid (RNA) DEGs, including known TME markers such as platelet-derived growth factor receptor alpha (PDGFRA). Tissue microarray (TMA) analyses of resections showed that deserted subTMEs had increased collagen content and B-cell marker (cluster of differentiation 20 or CD20) expression, whereas the reactive subTMEs had higher staining for cancer-associated fibroblasts (CAF), macrophages, T-cells, and endothelial cells. Of clinical interest, the co-occurrence of sub-TMEs in 53% of resections was a poor prognostic variable, as was the presence of a ‘deserted subTME’ due to its protective effect against Gem, GnP, and FFX. Though the effect of the TME subtype on the efficacy of anti-stromal agents has not yet been investigated, several other studies have highlighted variations in stromal composition and assigned a prognostic significance [89,90]. Moffitt et al. (2015) [89] used primary and metastatic TMA data to digitally separate the gene expression between PDAC stroma and normal tissue samples. The samples were clustered into three groups: those with ‘activated stroma’, ‘normal stroma’, or ‘absent stromal gene expression’. Kaplan–Meier analyses showed that the ‘activated stroma’ group had worse prognosis, with a median survival time of 15 months and a 1-year survival rate of 60% vs. 24 months and 82% in the ‘normal stroma’ group.

It has since been suggested that pancreatic cancer stem cells (PCSCs) could be responsible for the stromal variation and subsequent effects on disease outcomes in PDAC [91]. As cluster of differentiation-44 (CD44) and epithelial-specific antigen (ESA) are PCSC cell surface markers, one study analysed PDAC surgical resection tissue (n = 93) and found that a ‘loose’ stroma with fewer fibroblasts was highest in CD44^+^/ESA^−^ (63%) and CD44^+^/ESA^+^ (50%) tumours and that a dense, fibroblast-rich stroma was highest in CD44^−^/ESA^−^ tumours. In a 20-month follow-up study, no local recurrence was observed in patients with dense stroma, with the highest rate of recurrence seen in loose stroma (7% [95%CI: 1–19%] at 1 year and 18% [95%CI: 6–35%] at 3 years) (n = 31), but this was not statistically significant (*p* = 0.230). Loose stroma was linked to shorter OS (median of 16.1 months [95%CI: 12.4–32.8] vs. 48.5 [95%CI: 16.0–NR]) as compared to dense stroma (*p*  =  0.025). After adjusting for nodal status, this was not significant (*p* = 0.061). Despite this, the data suggest that stemness is linked to a loose stroma and worse outcomes in PDAC, suggesting that the stem cell compartment may dictate features of the stroma through differentiation of its components. Taken together, this suggests a need for patient stratification based on stromal heterogeneity, though in the context of PDT it is unclear what effect this would have.

## 3. PDT Mechanism and Clinical Progress

**Figure 3:**
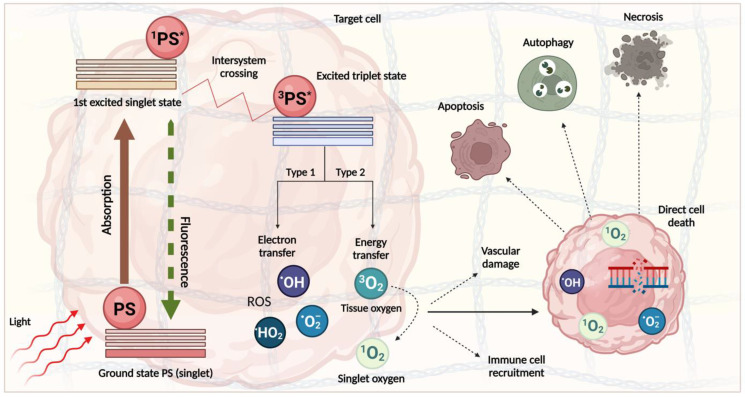
The mechanism of action PDT. After the photosensitiser (PS) is taken up by the cancer cell, light is delivered to the treatment area and absorbed by the PS, which adopts an excited singlet state (^1^PS). The PS is transferred to an excited triplet state (^3^PS) through the process of intersystem crossing and transfers an electron to biological molecules to form free radicals, including the hydroxyl radical (OH), hydroperoxyl radical (HO_2_), or the superoxide anion radical (O2^−^) (Type 1 reaction), or transfers energy directly to tissue oxygen, generating unstable singlet oxygen (^1^O_2_) (Type 2 reaction). Inside the cell, these highly reactive molecules lead to oxidative stress and DNA damage, rendering the cell susceptible to several forms of death, including apoptosis, necrosis, or autophagy. This figure was created on Biorender.com (accessed on 5 January 2023).

PDT is a form of photoactive therapy that elicits a cytotoxic effect via the release of reactive oxygen species (ROS) using a light-activated photosensitiser (PS). The treatment is somewhat specific owing to the partially selective accumulation of the drug within the target tissue and the targeting of the light to the tumour and its direct surroundings. The attractiveness of this approach has led to its use for several diseases, including oesophageal, mouth, and lung cancer [92,93,94]. PDT has proved efficacious in destroying advanced PDAC tumours in preclinical and clinical trials, though investigation is ongoing (Table 1 and Table 2). The mechanism of PDT is well understood. Once the PS molecule is irradiated with a light wavelength corresponding to its absorption spectrum, it undergoes excitation, and the reaction proceeds down one of two routes (Figure 3). The type 2 reaction, where energy is transferred to a ground-state triplet oxygen molecule to form highly reactive singlet oxygen, is most common amongst currently approved PS molecules. This then exerts its effect on the target tissue via the introduction of oxidative stress and the subsequent destruction of various cellular components [95]. The damage directly initiates cytotoxic death via several forms, including apoptosis, autophagy, and necrosis, though other less common modes of death have been noted [96]. PDT also has two indirect effects: the instigation of a ROS-initiated inflammatory response causing an immune reaction and the injury of vascular endothelial cells leading to tumour vessel damage and the obstruction of blood flow. Importantly, the blocking of this flow is dependent on the timing between the drug and light applications (the drug–light interval or DLI). A shorter light application time following drug application favours this process. These responses play a significant role in light-mediated tumour destruction, with many protocols now being modified to enhance them further [97,98,99]. However, for PDT-mediated stromal depletion, there are practical considerations when targeting vascular and immune cell components owing to the diverse roles and heterogeneity of the PDAC stroma.

The benefits of PDT include its ability to alter specific cellular targets depending on how the therapy is used. Factors such as the type of PS, light dose, light–dose interval, and drug dose–light dose interval all have an impact on the cytotoxic effect [100,101]. For example, different photosensitisers tend to localise within different organelles; this determines the type of cell death due to the initiation of different signalling pathways [102,103]. The PS can also be modified to increase targeting to the tumour vasculature, rather than the cancer cells [99,104]. Whilst these data have previously only been used to improve the efficacy of PDT and decrease its off-target effects, this control could be leveraged in the context of stromal depletion to achieve a balance between its destruction and maintenance.

### Clinical Application of PDT for PDAC

PDT using mTHPC (meta-tetra(hydroxyphenyl)chlorin) first entered clinical trials for PDAC in 2002 [105]. The first study established the efficacy and technical feasibility of percutaneous, computerised tomography (CT)-guided light delivery for PDT in unresectable PDAC. A median survival time of 9.5 months after PDT was recorded. The next trial used a shorter-acting PS, Verteporfin, which enabled better penetration of light to the tissue [75]. A year later, the first phase I trial of the endoscopic ultrasound (EUS)-guided, flexible laser light catheter PDT was performed, and no negative effects were observed [106]. A 2018 trial confirmed these results using Porfimer Sodium (Concordia Laboratories Inc, St Michael, Barbados) [76]. Patients were given Gem/GnP following PDT, and the median OS was 11.5 months. The most recent 2021 study of EUS-guided Verteporfin-mediated PDT had a median OS of 6.8 months [77]. It is difficult to draw conclusions regarding survival advantages from small, nonrandomised studies with few participants and a variable treatment history. In some instances, subsequent oncological treatments were given. Though larger, randomised studies are required to address this, the clinical trials so far have suggested a useful role for PDT as a (neo)adjuvant in PDAC, or even as a single therapy. In addition, all the studies were of patients with LA disease who either did not respond to previous CT or were unsuitable for surgery. It is only within this patient population that PDT has so far been examined, though its use for stromal depletion may be more useful for different stages of PDAC.

**Table 1:** Characteristics of in vivo preclinical PDT studies for PDAC.

Study	PS	PS Delivery	Light Source Delivery	Targeted or Untargeted PS	PS Dose	Light Dose	Drug–Light Interval	Cell Line or Species	Samples (n)	PDT Effect	Adverse Effects	Outcomes	Ref
Schroder et al., 1988	^125^I-labeled DHE	IV	Bare laser	Untargeted	4 mg/kg	40 J/cm^2^ (fluence)	3 h	Syrian golden hamster	Distribution study (33), PDT effect study (7)	Necrosis	Duodenal and jejunum perforation (3), death (4), haemorrhage (3), necrosis of liver and spleen (4).	PDT led to necrosis, haemorrhage, and inflammation of the tumours. Of the surviving 2 animals, there were no remaining areas of regular tumour.	[107]
Nuutinen et al., 1991	AIS2Pc	Intra-IVC	Bare fibre on tissue surface	Untargeted	1–5 μmol kg^−1^	50 J/cm^2^ (fluence) 50 mW/cm^2^ (intensity)	48 h	Female Syrian golden hamster	12	Necrosis	At 5 μmol kg^−1^: duodenal perforations (2), gastric ulcer (2), bile leakage (2), necrosis of liver parenchyma. At 1 μmol kg^−1^: a duodenal perforation (1).	With 5 μmol kg^−1^ pancreatic, necrosis was observed. With 1 μmol kg^−1^, no photodynamic effect was seen in the pancreas (laser placed on pancreas).	[108]
Chatlani PT et al., 1992	AIS2Pc	Intra-IVC	Bare fibre on tissue surface	Untargeted	5 mg/kg	Normal pancreas: 50, 100, and 200 J/cm^2^ (fluence). Tumour: 50, 25, and 12.5 J/cm^2^ (fluence). 25 or 50 mW/cm^2^ (intensity).	48 h	Female Syrian golden hamster	N/A	Necrosis	At 200 J/cm^2^, the normal pancreas demonstrated evidence of damage. In control animals, light doses > 50 J/cm^2^ led to gastric lesions.	All treated tumours showed evidence of coagulative haemorrhagic necrosis. Areas of necrosis extended beyond 5 mm in diameter (to the end of tumour nodules) when 25 or 50 J/cm^2^ doses were used.	[109]
Evrard S et al., 1994	Pheophorbide A	IV	Bare fibre on tissue surface	Untargeted	3 or 9 mg/kg	35, 75, and 100 J/cm^2^ (fluence). 120 mW/cm^2^ (intensity) Halogen light use at 216 or 432 J/cm^2^ (fluence).	24 h	Rat	Experimental (9), controls (36)	Necrosis	Duodenum injury	Six rats in experimental group were cured in 120 days. All control rats died in 35 days.	[110]
Regula J et al., 1994	5-(ALA)	IV or oral	Bare fibre on tissue surface or external irradiation using light-integrating cylindrical applicator	Untargeted	IV (200 mg/kg^−1^), oral (400 mg/kg^−1^)	Bare fibre: 50 J/cm^2^ (fluence) and 50 mW/cm^2^ (intensity) Cylinder application: 50 J/cm^2^ (fluence) and 100 mW/cm^2^ (intensity)	4 and 5 h	Syrian golden hamster, pancreatic cancer line PC-1	Experimental (9), controls (4)	Necrosis	Carcinomatosis (10), ascites (10), duodenal infiltration (2), malnutrition (2), and death (8)	Necrosis was evident in all 13 tumours. Smaller tumours showed complete necrosis. PDT-induced necrosis extended from the borders of the tumour. Mean survival time of controls was 42 days: animals treated with 400 mg/kg^−1^ oral ALA and 50 J/cm^2^ light using clinical applicator survived longer (*p* > 0.02). One mouse survived 116 days before being killed.	[111]
Mikvy P et al., 1996	mTHPC	IV	Bare fibre on tissue surface	Untargeted	1 mg/kg^−1^ for in vitro studies and 0.1 or 0.3 mg/kg^−1^ in vivo	50 J/cm^2^ (fluence) 50 mW/cm^2^ (intensity)	2 or 4 days	Syrian golden hamster	20	Necrosis	Free or sealed duodenal perforation (13), partial reversible bile duct obstruction (7)	Maximum necrosis seen 3 days after PDT. Lesions up to 4 mm in pancreas. Fractionating light dose increases lesion size by 30%.	[112]
Mikvy P et al., 1997	mTHPC	Intra-IVC	Bare fibre on tissue surface	Untargeted	0.1 or 0.3 mg/kg^−1^	50 J/cm^2^ (fluence) 50 mW/cm^2^ (intensity)	2 or 4 days	Syrian golden hamster, pancreatic cancer line PC-1	16	Necrosis	Duodenal perforation (3), bile duct obstruction (4), and duodenal diverticula (2) in experiments with higher dose (0.3 mg/kg^−1^) and fractionated light delivery	Tumour necrosis was highest 3 days post-PDT (maximum zone 12.4 mm in diameter using light fractionation). Treated tumours were histologically haemorrhagic in the centre and surrounded by inflammatory infiltrate.	[113]
Chan H-H et al., 2004	Porfimer sodium (Photofrin)	IV	Percutaneous light catheters (EUS)	Untargeted	4.4. mg/kg^−1^	50 J/cm^2^ (fluence) 0.4 W/cm^2^ (intensity)	24 h	Pig (farm, swine)	3	Necrosis	Gross ecchymosis on pancreas surface (1), inflammation, slight haemorrhage (3)	Necrosis of 3.6 mm^2^ can be achieved with 50 J/cm^2^ light. One hundred percent necrosis could be achieved in the pancreas. EUS-guided, low-dose PDT for ablation of the pancreas is feasible and safe.	[114]
Tangutoori et al., 2016	BPD (Visudyne)	IV	Transcutaneous	Targeted (anti-VEGF mAb bevacizumab)	0.5 mg/kg	75 J/cm^2^ (fluence) 100 mW/cm^2^ (intensity)	1 h	AsPC-1, nude mice	Total animals used 54.	Necrosis	Temporary oedema and erythema. Weight loss was minimal, and all groups were within standard limits of toxicity.	Nanoliposomes (nanoPAL) achieved significantly enhanced tumour reduction. No tumour regrowth for 34 days after treatment in all treated mice. Thirty-three percent of nanoPAL mice had complete response.	[115]
Li et al., 2017	5-ALA (PpIX), Cy5.5	IV	Direct laser irradiation	Targeted (U11 peptide)	2 pmol per mouse	50 mW cm^−2^ (intensity)	24 h	PANC1-CTSE, nude mice	Total animals used 96 (24/group)	Apoptosis	None reported	Both PDT and PTT alone can mediate tumour reduction using targeted NPs. PDT + PTT is most effective, as indicated by a higher number of apoptotic cells post-treatment. PDT + PTT leads to a better survival rate (just 1 death over 40 days).	[116]
Obaid et al., 2019	BPD (Visudyne)	IV	Direct NIR irradiation	Targeted (anti-EGFR mAb)	0.5 mg/kg BPD equivalent	40 J/cm^2^ (in vitro), 150 J/cm^2^ (in vivo) (fluence) 150 mW/cm^2^ (intensity in vitro) 100 mW/cm^2^ (intensity in vivo)	12 h	PaCa-2, PCAF, mice	24 total	Necrosis	Mice treated with targeted constructs remained healthy following PDT, whereas mice treated with untargeted constructs developed cachexia, weight loss, and moribundity.	Targeted low-dose PDT induced substantial necrosis in tumour tissues (3-fold increase in necrotic area 72 h post-PDT). Targeted PDT reduced tumour collagen density 1.5-fold.	[117]
Quilbe et al., 2020	A novel PS named ‘PS2′ (Pyro-PEG-FA) bound to folic acid (WO2019 016397-A1)	Intraperitoneal injection	Homogenous illumination (in vitro), Extracorporeal fractionation (in vivo)	Targeted (PS-FOL/PS2)	100 µL of PS solution at 1 mg/mL	3.6 J/cm^2^ (in vitro), 29.7 J/cm^2^ (in vivo) (fluence) 1 mW/cm^2^ (intensity in vitro) 11 mW/cm^2^ (intensity in vivo)	N/A	Capan-1, Capan-2, MiapaCa-2, Panc-1, SCID mice	8	N/A	None reported	The PS preferentially binds to the membrane of pancreatic cancer cells and is internalised (intracellular labelling detected). Mice subjected to PDT showed tumour growth decrease over time after illumination. PS-FOL/PS2 significantly limits tumour growth in SCID mice.	[118]
De Silva et al., 2020	BPD (Visudyne)	N/A	N/A	Untargeted	N/A	N/A	N/A	C57BL/6 mice	N/A	N/A	N/A	Infiltration of B- and T-cells to the tumour site was observed 1 h–5 days post-PDT. Activated T-cells and DCs were observed in the spleens of PDT-treated animals.	[119]
Sun et al., 2021	Pyropheophorbide A (PPa)	IV	Direct laser irradiation	Targeted (HA)	5 mg/kg	200 mW/cm^2^ (intensity)	24 h	Panc02 murine pancreatic tumour cells, C57BL/6 mice	Total animals used 25 (5/group)	Apoptosis	None reported	HA targeted Ppa-NPs activate CD8T^+^ cells in vivo. PDT-mediated tumour volume reductions and survival rates were highest with HA-targeted NPs containing Ppa and JQ1 (a BRD4 inhibiting drug). Apoptotic cell death was observed with NPs following PDT.	[120]
Vincent et al., 2021	BPD (Visudyne)	N/A	Direct laser irradiation	Untargeted	0.5 mg/kg	75 J/cm^2^ (fluence) 100 mW/cm^2^ (intensity)	1 h	BxPC-3 human PDAC cells, nude mice	5 total (2 for PDT experiment)	Necrosis	None reported	PDT priming mediated a 13% collagen reduction and significant regions of necrosis in fresh tissue samples.	[121]
Obaid et al., 2022	BPD (Visudyne) in liposomes (lipidated BPD or BPD-PC)	IV	Bare fibre on tissue surface	Targeted (anti-EGFR mAb)	0.25, 0.50, or 0.75 mg/kg^−1^ BPD equivalent	In vitro—20 J cm^2^. In vivo—0, 50, 100, or 150 J cm^2^ (fluence) In vivo—100 mW/cm^2^ (intensity)	90 min or 12 h	MIA PaCA-2 cell line, patient-derived CAFs, athymic male Swiss nu/nu mice	4–8 per experiment	N/A	None reported	Targeted liposomes induce 90% tumour growth inhibition at 8.1% of the equivalent dose of nanoliposomal formula. Photoactivation is ineffective without EGFR targeting. Targeted liposomes reduce collagen density by >90% and increase collagen nonalignment by >10^3^-fold.	[122]
Liu et al., 2022	Protoporphyrin IX (PpIx)	Intratumoural injection	Bare fibre on tissue surface	Untargeted	5.6 mg/kg	1 W/cm^2^ (intensity)	5 h	Panc-1 cells, nude mice	25 total (5 per group)	Apoptosis	None reported	Significant apoptosis of PSCs was observed in the PDT group. Reduced ECM deposition (including collagen and fibronectin) and downregulated expression of TGF-β and CTGF was observed in the PDT group. PDT resulted in >87% tumour volume reduction.	[123]
Yang et al., 2022	N/A	IV	Direct NIR irradiation	Au nanocage functionalised using collagenase and targeted using membrane coating	10 mg/kg	N/A	N/A	BxPC3, BALB/c mice, BALB/c nude mice	6	Apoptosis, necrosis, ECM degradation	None reported	Targeted and collagenase functionalised NPs exhibited the greatest extent of cell necrosis/apoptosis and highest inhibition of tumour growth. Survival of mice was significantly longer in this group.	[124]

Abbreviations: Ref, reference; IV, intravenous; DHE, dihematoporphyrin ether; AIS2Pc, disulphonated aluminium pthalocyanine; 5-ALA, 5-Aminolevulinic acid; mTHPC, meta-tetra(hydroxyphenyl)chlorin; BPD, benzoporphyrin derivative; Cy5.5, Cyanine5.5; PS, photosensitiser; NIR, near infrared; EGFR mAb, epidermal growth factor receptor monoclonal antibody; Au, silver; FOL, folate; ECM, extracellular matrix; NPs, nanoparticles; PDT, photodynamic therapy; Pyro-PEG-FA, Pyropheophorbide-Polyethylene Glycol-Folic Acid; BPD-PC, lysophosphocholine BPD conjugate; DCs, dendritic cells; CAF, cancer associated fibroblast; SCID, severe combined immunodeficiency disease; PSCs, pancreatic stellate cells; PCAF, pancreatic cancer associated fibroblast; CTSE, Cathepsin E; Intra-IVC, into the vena cava; EUS, endoscopic ultrasound; BRD4, bromodomain-containing protein 4; HA, hyaluronic acid; PPa; pyropheophorbide A; PpIX, protoporphyrin IX; TGF-β, transforming growth factor beta; CTGF, connective tissue growth factor; PTT, photothermal therapy; CD8T^+^, cluster of differentiation 8 T-cells; N/A, not available.

**Table 2:** Characteristics of clinical PDT studies for PDAC.

Study	PS Used	PS Delivery	Light Source Delivery	Targeted or Untargeted PS	PS Dose	Light Dose	Drug–Light Interval	Disease Staging	Number of Patients	Inclusion Criteria	Exclusion Criteria	PDT Effect	Side Effect	Survival	Ref
Bown et al., 2002	mTHPC	IV	Laparoscopy	Untargeted	0.15 mg/kg	20–40 J/cm^2^ (fluence) 100 mW/cm^2^ (intensity)	3 days	Stage 1–3 (UICC TNM)	16	Surgical suitability, Karnofsky status > 60% with anticipated survival of at least 3 months, attendance of follow-up	Ampullary cancers and those with cholangiocarcinoma, metastatic disease, previous specific treatment	Necrosis	Early: duodenal necrosis, CBD-duodenal fistula, necrosis around stents and ampulla, ulceration. Late: tumour ingrowth in stent, stenosis	Median 9.5 months	[105]
Gerdes et al., 2004	Porfimer sodium (Photofrin)	IV	Percutaneous or endoscopic	Untargeted	N/A	N/A	2 days	LA or advanced	4	Age > 18, primary carcinoma of pancreas, bile duct, gallbladder, or metastatic bile duct disease, unresectable disease, refusal of surgery if resectable, Karnofsky status 50–100%, bilirubin at least 2 mg/dL	Chemotherapy in last 4 weeks, concurrent RT or brachytherapy to the abdomen, administration of prior or concurrent experimental or investigational drugs	N/A	N/A	No results posted	[125]
Huggett et al., 2014	Benzoporphyrin derivative (Verteporfin)	IV	Laparoscopy	Untargeted	0.4 mg kg^−1^	5–40 J/cm^2^ (fluence) 150 mW/cm^−1^ (intensity)	60–90 min	LA	15	Unsuitable for surgical resection, adequate biliary drainage, EOCG performance status of >2	Porphyria, LA disease involving > 50% circumference of the duodenum or a major artery within treatment area, metastatic disease	Necrosis	Abdominal pain (3), rise in amylase (1)	Median 15.5 months	[75]
Choi J-H et al., 2015	Chlorin e6 type derivative (Photolon)	IV	EUS	Untargeted	2.5 mg/kg	100 J/cm^2^ of diffuser length (fluence) 300 mW/cm^2^ (intensity)	3 h	LA	4	LA disease with no metastasis after conventional CRT	Porphyria, major vessel in treatment area, ECOG > 2	Necrosis	None reported	No long-term follow-up	[106]
DeWitt et al., 2019 NCT01770132	Porfimer sodium (Photofrin)	IV	EUS	Untargeted	1–2 mg/kg	50 or 100 J/cm^2^ (total doses 150 or 300 J/cm^2^) (fluence) 400 mW/cm^2^ (intensity)	40–50 h	T3N0 (1), T3N1 (3), T4N0 (3), T4N1 (5)	12	Aged 18–75, unresectable LA disease, Karnofsky status > 70% with anticipated survival of at least 3 months	Metastatic disease, previous CRT, gastric or duodenal ulcers, cystic component > 25% tumour volume, ascites, bowel fistula, portal HT, bulky celiac adenopathy (>2.5 cm diameter), uncorrelated coagulopathy, renal insufficiency, etc.	Necrosis	Sunburn (1), nausea (1), photosensitivity (1), skin hyperpigmentation (1), fatigue (1)	Median 11.5 months	[76]
Hanada et al., 2021	BPD (Verteporfin)	IV	EUS	Untargeted	0.4 mg/kg	50 J/cm^2^ (fluence) 150 mW/cm^2^ (intensity)	60–90 min	T3 (5), T2 (2), T1 (1)	8	LAPC with adequate biliary drainage	Metastatic disease, disease involving > 50% duodenal or major artery circumference, recent treatment with curative intent	Necrosis	None reported	As of November 2020, 7 patients died with a median survival time of 6.9 months from procedure date. Data collection ongoing	[77]
Chandrasekhara et al., 2022	BPD (Verteporfin)	IV	EUS	Untargeted	0.4 mg/kg	50 J/cm^2^ (fluence)	1 h	LA or advanced	30 (still recruiting)	Age > 18, measurable disease defined by RECIST, ECOG of 0, 1, or 2, estimated life expectancy > 12 weeks, adequate biliary drainage	In LA patients, metastatic disease other than lung or liver; lung metastases with greater than 3 lesions or any lesion greater than 5 cm, porphyria, pregnant or breastfeeding, LA disease involving > 50% circumference of duodenum or artery in treatment area, recent treatment with curative intent, history of haemorrhagic diathesis or coagulopathy, other malignancy or systemic disease	Necrosis	None reported (data collection ongoing)	Data collection ongoing	[126]

Abbreviations: Ref, reference; mTHPC, meta-tetra(hydroxyphenyl)chlorin; IV, intravenous; EUS, endoscopic ultrasound; UICC TNM, Union for International cancer control Tumour Node Metastasis standard for cancer staging; LA, locally advanced; portal HT, portal hypertension; LAPC, locally advanced pancreatic cancer; EOCG, Eastern Cooperative Oncology Group; RT, radiotherapy; CRT, chemoradiotherapy; RECIST, response evaluation criteria in solid tumours; PDT, photodynamic therapy; PS, photosensitiser; BPD, benzoporphyrin derivative; N/A, not available.

## 4. Role of PDAC Stroma and Implications for PDT

**Figure 4:**
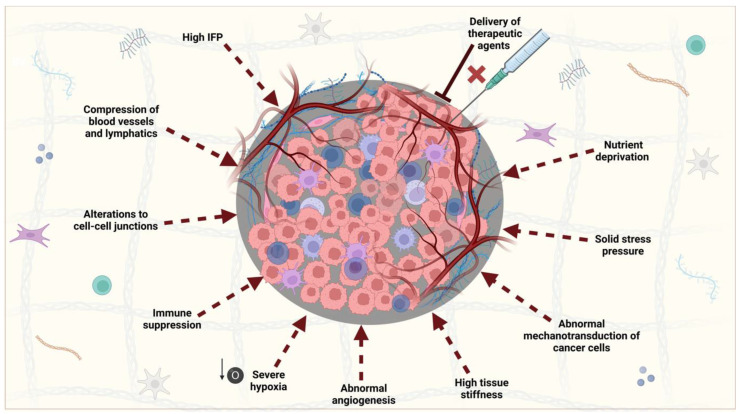
The overall physical and biophysical effects of the PDAC stroma on the tumour and its TME. The unique stroma in PDAC has a broad range of implications that prevent the delivery of therapeutic agents, mainly via their effects on blood vessels. This includes high levels of IFP, solid stress, and tissue stiffness. Adding to this effect is the unusual morphology of the vessels, owing to abnormal angiogenesis characteristic of the disease. The result of these features creates a harsh environment where PDAC cells are subject to nutrient deprivation, severe hypoxia, and immune suppression. This contributes to tumourigenesis and is further enhanced by the production of cytokines which interfere with PDAC cell–cell junctions and their mechanotransducive properties. As a result, PDAC cells gain a more migratory and invasive phenotype. IFP, interstitial fluid pressure. This figure was created on Biorender.com (accessed on 5 January 2023).

The dense peri-tumoral stroma in PDAC imparts an array of molecular and biophysical effects on the tumour and its constituents (Figure 4). Together, these are responsible for the restriction of the delivery of treatments to the site, which is thought to be one of the reasons for poor survival. Whilst this cancer protective effect could be addressed using PDT-mediated stromal depletion, these challenges are extended to the successful clinical implementation of PDT for two reasons. Firstly, the desmoplastic reaction creates a dense environment with high compressive stress that causes blood and lymph vessels to be occluded; analysis of MMTV-M3C breast tumours showed that the fraction of perfused vessels decreased with tumour size and an associated higher level of stress [127]. This is contributed to the hygroscopic nature of hyaluronic acid (HA), which causes the localised trapping of water [128]. One study investigated orthotopic MiaPaCa2 PDAC tumours and found that the swelling stress was 16.01 ± 1.66 mmHg (2.13 ± 0.22 kPa), whereas in healthy tissues this was much lower, usually around zero [129]. This was linearly proportional to the ratio of the HA/collagen area fraction (n = 5) and could be decreased experimentally by hyaluronidase, an enzyme that breaks down HA. Analysis of perfused blood vessels as a function of tumour swelling showed that there was an exponential decay relationship in vivo, suggesting their physical occlusion. Once the vessels are occluded, IFP, which is zero in normal tissues, rises and equilibrates with microvascular pressure as the extracellular fluid cannot drain [130]. Analysis of PDAC tumour IFP levels in genetically engineered *KPC* mice, which act as a clinically relevant model of PDAC, have been reported as being as high as 76 ± 4.2 mmHg vs. −0.73 ± 0.6 mmHg in the normal mouse pancreas [131]. Whilst abnormal angiogenesis usually contributes to leaky vasculature in many solid tumour types, where drugs are able to exit the bloodstream more easily via the enhanced permeability and retention (EPR) effect, the occlusion and eventual collapse of blood vessels in PDAC as a result of the high pressure imposed by the stroma prevents this [128]. As the movement of macromolecules into the tumour is prevented, drug delivery is restricted. A 2022 study found that serum concentrations of ‘free’ doxorubicin (Dox) in subcutaneous mouse PDAC tumours were just 0.8 µg/mL 15 min after administration, and the drug was cleared entirely by 4 h [132]. The same challenges are faced with the entry of PS agents in PDAC [133]. Secondly, the lack of oxygen transport makes the TME hypoxic, which restricts the efficacy of PDT by limiting light penetration as well as the actual cytotoxic effect (as this is dependent on the presence of molecular oxygen). The hypoxia also feeds into the desmoplastic reaction, which perpetuates the effect further [134]. Careful depletion of the PDAC stroma by PDT might allow these obstacles to be overcome (see Section 6).

## 5. Pleiotropic Nature of the PDAC Stroma

Many of the stromal constituents in PDAC display significant heterogeneity, not just in their composition between and within tumours, but also in their function. Specifically, it is now appreciated that the PDAC stroma has both pro- and anti-tumour effects. The data currently support the growing observation that the stroma helps restrain the tumour epithelial component, acting to suppress local invasion. One study investigated this by manipulating the levels of collagen in an in vivo model of pancreatic cancer [135]. As bone morphogenic protein 1 (BMP1) promotes collagen deposition, the study first confirmed that BxPC3 (human pancreatic cancer cells) overexpressing BMP1 (lenti-BMP1) could enhance collagen subunit α1(I) protein levels in vitro. They then established that BxPC3 cells knocked down for *COL1A1* expression (shCOL1A1) did not enable collagen subunit α1(I) protein production. Collagen deposition from PDAC tumour cells was then shown to suppress tumour growth in vitro. In lenti-BMP1 cells, BxPC3 cell growth, measured by 1/doubling time (hours), was significantly reduced compared to untreated control cells (~0.035 vs. ~0.04). Accordingly, shCOL1A1 cells reversed this growth suppression (~0.044). In vivo, lenti-BMP1 primary tumours were smaller than those of the controls (~100 mg vs. ~210 mg (*p* < 0.01)) and shCOL1A1 (~500 mg) or lenti-BMP1 + shCOL1A1 (600 mg) tumours. Therefore, the presence of collagen is associated with smaller tumours. Metastasis was also significantly reduced in lenti-BMP1 tumours compared to the controls (~0.02 vs. ~0.3 lung met load/g tumour (*p* < 0.01)). Both shCOL1A1 and lenti-BMP1 + shCOL1A1 groups also had much greater lung metastases (~3 and ~4, respectively). The data suggest that cancer cell-derived collagen acts to prevent cancer growth and progression. In keeping with this, a recent 2021 study found that PDAC tumours with myofibroblast (MF) Col1 deletions had a reduction in desmoplasia-associated markers such as alpha smooth muscle actin (αSMA) and reduced biophysical stiffness in mice [136]. The tumours progressed much more quickly: 50% of the excised tissue represented pancreatic intraepithelial neoplasia (PanIN) compared to ~30% in mice without the deletion. It has been suggested that the tumour-suppressive effect of collagen may in part be due to its mediation of an increased immune response through allowing T-cell infiltration [84]. This study analysed the spatial distribution of cytotoxic T-cells in PDAC patient tissue samples using computational imaging: the authors discovered a larger collagen-I presence in pericancer areas where T-cells were seen (~1500 collagen-I grey values within 20 μm of cancer cells vs. ~700 where T-cells were not present, *p* < 0.05). In addition, analysis of TMA cores showed a positive correlation between collagen-I deposition and the percent of cytotoxic or CD4 T effector cells per patient. Though the mechanism of increased T-cell infiltration in the presence of collagen is yet to be fully elucidated, the previous authors associated the deletion of MF Col1 with the recruitment of MDSCs via C-X-C motif chemokine 5 (CXCL5) upregulation [136]. MDSCs are known to suppress T-cell activation and function; thus, their negative association with collagen may explain its tumour-suppressive effect in PDAC.

The same pattern of conflicting tumour-promoting and tumour-suppressing activity can be seen in the PDAC CAF population. An in vivo study using transgenic mice with aSMA^+^ MF deletions showed that the depletion of these cells led to more invasive tumours [137]. Though cancer-associated myofibroblast (mCAF) cells are usually associated with tumour progression, their deletion in the study led to various tumour-promoting events, such as enhanced PDAC migration, hypoxia, and a cancer stem cell-like phenotype. Another study showed that deletion of sonic hedgehog (Shh), the ligand that activates a number of growth and tumourigenic pathways, gave rise to reduced stromal content in a mouse model of PDAC, including a decreased mCAF population; the study associated this with more aggressive disease: mean survival was significantly shorter in these mice compared to mice without the deletion (19.2 ± 5.27 vs. 6.5 ± 2.7 days, *p* < 0.001) [138]. Potential theories for the tumour-suppressive role of mCAFs include the possibility that they may be involved in the release of differentiation cues such as BMPs [139] or that, as with collagen, they can increase the immune response by suppressing regulatory T-cell (Treg) infiltration [137]. As the tumour-supressing role of mCAFs was established in the above studies, efforts have been underway to understand how this population of cells in PDAC carries out contradictory functions.

For some time, a dynamic population of CAFs in PDAC has been appreciated [140,141,142], with distinct myofibroblast, inflammatory (iCAF), and now metabolic (meCAF)- [143] and antigen (apCAF)-presenting [144] subtypes. Studies are now using a variety of sophisticated models to explore the mechanisms of CAF differentiation, with particular focus on their original source and how this gives them different roles [145,146,147,148]. One study has also identified variations in the transcriptional and metabolic activity between fibroblast subtypes in PDAC using single-cell sequencing data [149]. For example, mCAFs were associated with pathways related to focal adhesion and ECM–receptor interaction, as well as to the tricarboxylic acid (TCA) cycle and oxidative phosphorylation (OXPHOS), whereas iCAFs were associated with cytokine–cytokine receptor interaction pathways and glycolysis. Presumably, under hypoxic conditions the fibroblasts would adopt an iCAF phenotype, which illustrates their ability to react to signals from the TME. Importantly, studies investigating PDAC subTMEs have found that different CAF subpopulations might preferentially localise to distinct tumour regions: single-cell analyses and functional characterisation of CAFs from 13 patient samples showed that the growth patterns of subTME CAF monolayers reflected that of their originating subTMEs, with ‘reactive’ subTME-derived CAFs being more motile and ‘deserted’ subTME-derived CAFs growing faster [88]. The data suggest that subTMEs comprise a highly complex community of CAFs with various differentiation states and associated functions.

These data present a new challenge in the application of PDT for stromal targeting in PDAC as extreme depletion may promote tumour progression and therefore undermine patient survival. In the context of PDT-mediated stromal depletion, this may also restrict its ability to resensitise the cells to any subsequent treatment. In fact, although variations in stromal composition and having a ‘stroma activated’, ECM-rich, or desmoplastic transcriptional signature are associated with worse prognoses [89,150,151], other studies have found that ECM and collagen-abundant stroma yield better prognoses [152,153]. For example, in a 2019 study that analysed the RNA of PDAC samples from 125 patients, ‘basal-like/ECM-rich tumours’ were associated with a poorer prognosis compared to ‘classical/immune-rich tumours’ (hazard ratio 3.76 vs. 2.11) [150]. Similarly, in an analysis of resected PDAC samples from 309 patients between 1996 and 2010, having a ‘stroma activated’ tumour with high stromal content had a much poorer median OS of 20.2 months vs. 43.1 months in ‘pure classical’ and 37.4 months in ‘immune classical’ tumour subtypes [151]. However, the Grünwald et al. (2021) study that looked at subTMEs in early-stage resected PDAC samples associated ‘reactive’ subTMEs, which are characterised by very little ECM and collagen content, with shortened disease-free survival compared to ‘deserted’ (ECM high) subTMEs, though this was not significant (*p* = 0.07) [88]. The data suggest that it is possible that the dense ECM could restrict the growth of the tumour and therefore yield better survival outcomes; however, they also suggest that this is not the case for all patients. Instead, the findings point to a more complex picture where the dynamic interaction between stromal constituents and PDAC cells dictates various outcomes that vary based on disease states and specific molecular features.

## 6. The Effect of PDT on Stromal Components

**Figure 5:**
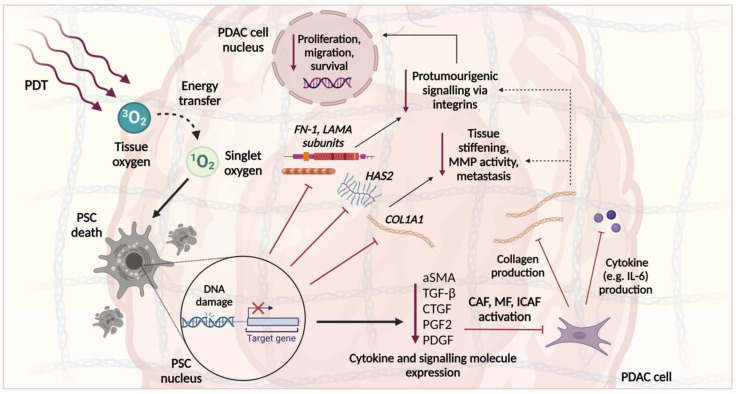
Proposed destruction of PSCs by PDT and subsequent effects on the PDAC stroma. The PDT-mediated production of highly reactive singlet oxygen mediates PSC death via the disruption of its organelles and various cellular constituents, such as lipids and proteins. The cellular death that results from this damage, as well as direct damage to the DNA material in the nucleus, causes the halting of expression and deposition of PSC products. This includes the ECM molecules collagen, HA, laminin, and fibronectin. As a result, the associated desmoplastic and pro-tumourigenic signalling routes are restricted, which reduces the proliferation, migration, and survival of the PDAC cells through altered nuclear gene expression. Destruction of PSCs also reduces their production of various signalling molecules and cytokines such as TGF-β. In turn, CAF activation is prevented, as is their ability to produce further ECM components and cytokines. This enhances the dampening of the desmoplastic reaction whilst also restricting adhesion to vessels and the formation of an immunosuppressive microenvironment. The figure was created on Biorender.com (accessed on 5 January 2023). PDAC, pancreatic ductal adenocarcinoma; PDT, photodynamic therapy; PSC, pancreatic stellate cell; FN-1, fibronectin 1; HAS2, hyaluronan synthase 2; αSMA, alpha smooth muscle actin; DNA, deoxyribonucleic acid; COL1A1, collagen type I alpha 1 chain; TGF-β, transforming growth factor beta; PGF2, prostaglandin F2; PDGF, platelet-derived growth factor; CAF, cancer-associated fibroblast; MF, myofibroblast; ICAF, inflammatory cancer-associated fibroblast; IL-6, interleukin-6; MMP, matrix metalloproteinase; CTGF, connective tissue growth factor.

A recent review by Karimnia et al. [154] has discussed the stroma and its effect on the application of PDT for PDAC, though the targets of PSD specifically, including the subsequent signalling effects, are yet to be defined. As the goal of PSD in PDAC is to physically disrupt the desmoplastic stroma to enable better subsequent treatment efficacy, the intended targets of the treatment are PSCs and CAFs, which together are responsible for the secretion of nearly all ECM components, including collagen, HA, prostaglandins (PG), and laminin (Figure 2). Because the cytotoxic effect cannot be provided by the PS directly to the structural ECM components, PSCs and CAFs are also appropriate targets because they possess endoplasmic reticulum (ER), mitochondria, and nuclei. The ROS-mediated damage to the organelles of PSCs and CAFs, as well as directly to their nuclear material, secondarily leads to the reduced ECM deposition, as shown in Figure 5. A reduction in the pro-tumourigenic signalling between the stromal constituents and cancer cells eventually also causes PDAC tumour death via a number of potential mechanisms, including apoptosis, necrosis, or autophagy. The feasibility of PDT-mediated damage to PSCs and CAFs has been shown in several pre-clinical studies, as described later.

### 6.1. Vascular Remodelling and Pruning

Another potential effect of PSD for PDAC is ‘vascular normalisation’, a technique where light is used to prune faulty and dysfunctional vessels by destroying their excess endothelial cells [155,156,157]. This increases the vessel pericyte coverage, which in turn reduces their leakiness and improves tumour perfusion. A recent 2019 study found that vascular-targeted low-dose PDT decreases tumour IFP through enabling better vessel pericyte coverage via the modulation of ras homolog gene family member A (RhoA) and myosin light chain (MLC) signalling in an in vivo model of malignant pleural mesothelioma [158]. More recently, elaborate systems have been designed to enhance this effect. In a 2022 study, an intelligent PDT nano delivery system was designed where PS-encapsulated nanoparticles (NPs) could respond to the hydrogen peroxide (H_2_O_2_) content of tumour endothelial cells [159]. LMWH-Ce6-luminol (LCL)-zinc oxide (ZnO) NPs normalised tumour vessels in vivo via the activation of nitric oxide synthase signals: pericyte coverage and vascular endothelial-cadherin (marker of strengthened adherent junctions) were increased and micro-vessel density was decreased by the treatment. Further analysis showed that LCL-ZnO NPs decreased metastasis and inhibited the immunosuppressive TME; CD8+ T-cell infiltration was improved 9 days after treatment with LCL-ZnO NPs compared to the PS alone (50 vs. 5 per field of view) (*p* < 0.001). Though studies of vascular normalisation for the PDAC stroma have not yet been reported, this technique would be particularly relevant in pancreatic cancers as dysfunctional vasculature and an immunosuppressive TME are features of the desmoplastic reaction. Better perfusion could promote the delivery of oxygen and PS to the tumour site to enhance the ROS-mediated cytotoxic effect, as well as help facilitate the subsequent delivery of cytotoxic agents.

On the other hand, this may be technically challenging as PDT also provides its cytotoxic effect through causing microvascular stasis and vessel destruction, resulting in nutrient deprivation and hypoxia [160,161,162]. However, this can be overcome by altering the delivery of the therapy. Data have shown that using a lower PS dose can help preserve vascular density and diameter, preventing extensive vessel damage, whilst a lower fluence rate or fractionating of the light delivery can reduce oxygen consumption and allow re-oxygenation of the tissue [163,164,165]. One study found that tumour oxygen levels were dependent on the fluence rate of Photofrin-PDT in vivo: the median tumour pO_2_ (mmHg) was a maximum of 12 with 30 mW/cm^2^, whereas this remained less than 5 using 75 or 150 mW/cm^2^ [166]. Though it is possible to limit treatment-induced hypoxia using cautious light delivery methods, additional strategies to increase tumour oxygen concentration will be important in the success of PSD for PDAC. One study found that Fe(III)-containing NPs could convert H_2_O_2_ to O_2_ in PANC-1 cells following PDT via a Fenton-like reaction [167]. Compared to untreated controls where no tumour volume change was observed, the NPs mediated a significant reduction (*p* < 0.05), suggesting that the NPs can increase the efficacy of PDT by overcoming tumour hypoxia. Another strategy would be to deliver oxygen to the site via microbubbles. Though not yet investigated for PDAC, a 2022 study of protoporphyrin IX-mediated PDT found that microbubbles could enhance tumour oxygenation from 22 to 50% in an in vivo model of breast cancer [168]. Overall, PSD for PDAC would need to be used carefully so as to destroy the relevant stromal components, in this case the dysfunctional vasculature, whilst sparing those that are important to preserve.

### 6.2. Targeting the Immune Cell Compartment

The PDAC stroma has many tumour-promoting effects, including the promotion and maintenance of an immunosuppressive TME. Generally, this lack of immunogenicity can be overcome by targeted agents. For example, a 2021 study showed that PDT combined with brodomain-containing protein 4 (BRD4) inhibitors promotes the immune response via the downregulation of programmed death ligand-1 (PD-L1) [120]. Another study showed that Visudyne-PDT led to the gradual increase in T- and B-cells at the tumour site up to 5 days after irradiation [119]. Cluster of differentiation 107a (CD107a) was upregulated in CD8+ T-cells following PDT, suggesting the enhanced priming of T-cells. As Interleukin 6 (IL-6) can both prevent and encourage the anti-tumour immune response, it is difficult to ascertain the benefits of its potential decrease by PSD for PDAC. Instead, the current preclinical PDT research for PDAC is focusing on combining the treatment with various immunotherapies that encourage the anti-tumour immune response in a more controlled way, including the regulation of specific molecules such as PD-L1, as described previously. However, these immunotherapeutic agents could be used alongside PSD to enhance both strategies. PSD would enable better delivery and efficacy of immunotherapies through reducing the dense and immunosuppressive TME, whilst the drug itself promotes the immune clearance of PDAC cells, preventing their contribution to the desmoplastic reaction and excessive ECM deposition.

### 6.3. Preclinical Investigation of PDT for Stromal Depletion in PDAC

Though the role of the stroma in tumour growth and progression has been appreciated since the early 2000s, the development of high-quality models that fully replicate the heterotypic TME has enabled more accurate pre-clinical investigation of PDT for stromal depletion in the last decade. In particular, 3D co-culture models that recreate the stromal environment and signalling contexts seen in vivo, especially for PDAC, are essential in predicting a clinically relevant PDT response due to the impact of these factors on cancer cell behaviour. Two commonly used 3D models are tumour spheroids, tightly bound cellular aggregates that are formed from transformed cells under nonadherent conditions, or gel embedding, which uses biological gels such as collagen gel to allow the spheroid to demonstrate a more accurate ex vivo cellular architecture [169]. The particularly dense stroma in PDAC inspired the first investigation of PDT for stromal depletion in 2012 [79]. Using a 3D fibroblast co-culture model, this initial study found that although the presence of fibroblasts had an unknown effect on the tumour response to PDT, their destruction following irradiation indicated that the therapy could be used to elevate the sensitivity of the surviving tumour nodules to subsequent interventions. Since then, the effect of stromal PSCs and fibroblasts on PDT has become clearer. They seem to provide a protective effect on PDAC cells, limiting the efficacy of benzoporphyrin derivative (BPD) and verteporfin-mediated PDT treatment [170,171]. Broekgaarden et al. (2019) [170] used microtumours, 3D cultures comprising PDAC cells, to investigate the mechanism of BDP-PDT; MIA PaCa-2 (MP2)-only microtumours had a half-maximal inhibitory concentration (IC_50_) value of 23.65 ± 1.86 J/cm^2^ compared to the values of 43.97 ± 2.63 J/cm^2^ in MP2 tumours cultured with CAF6 cells. The data suggested that CAFs could reduce the susceptibility of the microtumours to PDT via the provision of a protective effect. In sharp contrast, Karimnia et al. (2021) investigated the effect of Verteporfin-mediated PDT using 3D co-cultures and showed that cultures with PSCs or stromal fibroblasts (MRC5 cells) had an increased response to PDT; at 20 J/cm^2^, the PDT response (live vol/total vol) of CFPAC-1 (human pancreatic) cells was ~0.7 without PSCs and ~0.57 with PSCs (*p* < 0.05) [78]. The study also investigated the method of cell death following PDT, suggesting both apoptotic and necrotic mechanisms. Taken together, the destruction of PSCs and stromal fibroblasts by PDT enhances PDAC cell death via different mechanisms, including apoptosis and necrosis. Therefore, targeting the stromal component using PDT could be used to increase the anti-tumour effect of the treatment via these specific processes.

In addition to the destruction of PSCs and stromal fibroblasts, PDT has also shown an ability to physically disrupt the structural ECM constituents in PDAC. A 2021 study investigated the role of Visudyne-PDT in collagen depletion using mice injected with the human pancreatic cell line BxPC-3. Analysis of fresh tumour samples using ultraviolet (UV)-fluorescence imaging showed a 13% reduction in collagen content in PDT tumours vs. controls [121]. Moving forwards, the precise imaging of tumour collagen content, including fibre length, thickness, orientation, and the crosslinking profile, by such a technique will be a useful tool for the assessment of PDT for physical stromal depletion in PDAC. In addition, the destruction of the PDAC stromal collagen content using PDT can be enhanced by using a targeted PS. One study utilised a heterotypic PDAC organoid model comprising patient-derived CAFs to explore the functionality of Visudyne and Cetuximab-loaded epidermal growth factor receptor (EGFR)-targeted photoimmunoconjugates (PINs) [117]. The targeted PINs reduced collagen density 1.5-fold. In another study, the same authors used photoactivable multi-inhibitor liposomes (PMILs) containing Visudyne and Irinotecan targeted to EGFR [122]. Analysis of orthotopic PDAC tumour cryosections showed that the targeted PS mediated a 91.8% decrease in collagen density compared to untreated controls. Similarly, collagen nonalignment was increased > 10^3^-fold, and this was negatively associated with tumour burden and positively associated with PFS (≤0.005) as well as OS (*p* ≤ 0.05). Taken together, these results suggest the suitability of PDT for the depletion of the PDAC stroma.

### 6.4. Combined Treatment Strategies for PDT-Mediated Stromal Depletion in PDAC

Though the use of PDT alone has shown an ability to physically destroy various stromal constituents in PDAC, the focus has recently turned to addressing the fibroblast signalling pathways responsible for enabling the observed resistance to PDT in order to enable better treatment efficacy. Having confirmed that microtumours co-cultured with CAFs exhibit reduced treatment sensitivity to BPD-PDT and oxaliplatin, one study investigated the redox pathway as a potential player in this resistance due to the oxidative state of PDAC being previously linked to CAFs and treatment insensitivity [170,172,173]. Using a method for redox imaging based on nicotinamide adenine dinucleotide phosphate (NAD(P)H) and flavin adenine dinucleotide (FAD) autofluorescence, Broekgaarden et al. (2019) [170] found that the presence of fibroblasts in microtumour cultures significantly increased redox states and that this correlated to the upregulation of oxidative stress-induced survival proteins such as heme oxygenase 1 (HO-1), indicating the activation of the nuclear factor E2-related factor 2 (NRF2)-mediated antioxidant stress response. Subsequently, the OxPhos inhibitor metformin significantly enhanced the efficacy of both BPD-PDT and oxaliplatin in CAF tumours, suggesting that the CAF-induced treatment resistance could be overcome by reverting their increased oxidative state. Therefore, metformin could be used to enhance the destruction of CAFs by PDT, which in turn could resensitise the cells to subsequent chemotherapies, increase their efficacy, and permit the delivery of a less toxic dose. Similarly, as the vitamin D analogue calcipotriol (CAL) has previously been shown to reprogram PSCs (CAF precursors) into a non-cancer-associated, quiescent state by inducing a variety of epigenetic and transcriptomic changes [174,175], it has also been investigated in combination with PDT to enhance treatment efficacy [176]. Because C-X-C motif chemokine 12 (CXCL12) secretion by PSCs and CAFs is associated with tumour progression [177], the study aimed to measure this as an indication of the interruption of this pathway by the treatments. CAL + BPD-PDT facilitated a 49% reduction in CXCL12 secretion in PCAF cells compared to that of untreated controls; yet, treatment with CAL or PDT individually did not significantly reduce CXCL12 secretion. This suggests that the treatments act synergistically to suppress this pro-tumourigenic pathway by disrupting the CAF population. As the authors had previously found that Verteporfin-PDT could permeabilise the parenchyma and tumour-associated microvasculature to increase intra-tumoral chemotherapy delivery [178], PDT enhanced by CAL could be used to subtly interfere with tumour–stromal interactions in PDAC and thus improve its susceptibility to cytotoxic strategies.

As the metabolic contribution of CAFs in PDAC remains a subject of current investigation, future strategies based on this will likely enhance therapeutic approaches, including PDT. For example, a recent 2023 study discussed autophagy in PDAC cells as a mechanism for their survival in the harsh TME: through ferritinophagy, the autophagy of the iron storage protein ferritin, PDAC cells increase levels of free iron to promote their survival [179]. By inhibiting autophagy in PDAC cells using small interfering RNA (siRNA) against ATG5 (autophagy related 5), the authors demonstrated an altered mitochondrial function and a reduction in the labile iron pool (LIP). Accordingly, the autophagy inhibitor hydroxychloroquine (HCQ) has been investigated as a monotherapy for PDAC in clinical trials [180]. However, poor patient response led the above authors to investigate the role of the PDAC TME in this resistance. CAFs were co-cultured with siATG5 PDAC cells and, upon autophagy inhibition, the relative LIP was restored back to 100% of the siControl cells vs. ~15% of the siControls in the PDAC cell monoculture. Further investigation showed that this effect was likely due to an IL-6 mediated increase in iron exporter ferritin (FPN) by CAFs. This LIP compensation could also be prevented by the IL-6 receptor antagonist Tocilizumab; in CAF-PDAC cell co-cultures, the drug brought the relative LIP to ~75% vs. 100% in the siControls. In the monocultures, the relative LIP was ~10% vs. 100% in the siControls. Though further studies would be needed to establish the relevance of iron production by CAFs in PDT resistance, the study highlights the importance of metabolic PDAC-CAF crosstalk in PDAC survival and the need to utilise combination therapies such as HCQ that could target CAFs and increase any anti-tumour effect.

New investigation has found that PDT itself can encourage the activation of CAF signalling pathways, which introduces a further challenge surrounding its clinical implication for both PDAC cell destruction and stromal depletion. One study found that BPD-PDT promotes the secretion of hepatocyte growth factor (HGF) from CAFs, which in turn activates the MET survival pathway [181]. As MET signalling has previously been associated with tumour progression and treatment resistance in PDAC [182,183], the authors used the multi-kinase inhibitor cabozantinib to block MET phosphorylation to overcome this PDT-mediated effect. Subsequently, cabozantinib was shown to enhance the efficacy of PDT, as indicated by greater spheroid necrosis and fractional dead area, most notably at lower exposures (>10 J/cm^2^). However, this effect was observed whether fibroblasts were present or not, and cabozantinib was capable of abolishing MET phosphorylation and enhancing PDT regardless of MET expression levels. These findings are important as they suggest that PDT can be enhanced in conjunction with additional drugs such as cabozantinib. Because this effect was seen in the presence of fibroblasts in this study, their destruction is also unlikely to have any tumour-promoting effects, which is thought to be a reason for the failure of current stromal targeting strategies and a concern for PDT-mediated stromal depletion.

As described previously, one idea is to use PDT to enhance the delivery of additional therapeutic agents, particularly chemotherapeutic drugs, via the destruction and subsequent leakage of tumour-associated microvasculature. Using a chorioallantoic membrane model, one study showed that prospective ‘photodynamic drug delivery’ could be enhanced using the cyclo-oxygenase inhibitor aspirin [184]. Through delaying blood clot formation and enhancing the permeability of treated blood vessels, PDT with adjuvant aspirin could increase the extravasation of a fluorescent dye 2.4-fold. In practise, such combination therapies could improve treatment efficacy by enabling the local dosing of a chemotherapeutic drug whilst aiding in PSD through the destruction of blood vessels and the associated decrease in tumour pressure. This has been observed in several studies, including those on PDAC [117,185]. However, it is unclear how this might affect the process of the PDT-mediated vascular normalisation described previously, considering that this relies on reducing the leakage of vessel contents to enhance the perfusion of therapeutics. Ideally, the vasculature would be destroyed in a controlled manner to facilitate the temporary enabling of oxygen and drug delivery to the site. The use of low-dose or ‘sublethal’ PDT and nanomedicine-assisted delivery of PSs (see Section 7) will likely help in achieving a high level of controlled photoactivation where the necessary stromal components are preserved but the treatment efficacy is still maximised by the use of a combination of relevant strategies.

## 7. Recent Developments and Future Use of PDT for PDAC

For some time, molecular-targeted PDT, primarily in the form of photoimmunotherapy (PIT), has been investigated for PDAC in an attempt to increase the intensity and specificity of the ROS-mediated phototoxic effect on the cancer cells. Though many targets have been identified and several investigated at the preclinical stage for PDAC, including HA and EGFR (see Table 1), there are other routes to selective PDT-mediated PDAC destruction that are yet to be considered. For example, Lange et al. investigated targeted PDT using selective protease expression in prostate cancer [186]. By attaching the PS pheophorbide A to a polymeric carrier using peptide linkers that could be cleaved by urokinase-like plasminogen activator (uPA), a protease overexpressed in prostate cancer, the drug could selectively accumulate at the tumour site in vivo and facilitate effective tumour cell eradication. The treatment is thus more selective and off-target effects are minimised. One protease recently identified as upregulated in PDAC is kallikrein-related peptidase 6 (KLK6), which shows high expression in the TME, where it is suspected to have regulatory functions, including the degradation of ECM components [187]. KLK6 could be a useful target in specific light-mediated destruction in PDAC, particularly of the stroma itself.

Though the use of direct antibody–PS conjugates has so far been very promising in PDAC, the functionalisation of NPs for this purpose has become of particular interest for several reasons. In addition to providing a better and more specific cytotoxic effect via PDAC cell targeting, one benefit of PS-encapsulated nanocarriers is their small size, often in the range of 1–100 nm, which facilitates better drug distribution through the stroma. This is well evidenced in a 2019 study where stroma-rich heterotypic in vitro and in vivo PDAC models were used to investigate the mechanism of Cetuximab (anti-EGFR) photoimmuno-nano-conjugates (Cet-PINs) of Visudyne (BPD) [117]. The cell surface receptor EGFR was chosen for targeting as it is overexpressed in up to 85% of patients with PDAC. Cet-PINs exhibited a considerably higher binding affinity for MIA PaCa-2 + CAF organoids at all time points compared to untargeted constructs and could efficiently penetrate organoids after just 1 h of incubation. To confirm these results in vivo, a 0.5 mg/kg BPD equivalent of the Cet-PINs was administered into mice baring MIA PaCa-2 + CAF xenograft tumours. Analysis of the tumour sections showed that Cet-PINs could mediate substantial necrosis, with a statistically significant 3-fold increase in fractional necrotic area 72 h post-PDT, whereas no necrosis was observed for untargeted constructs. The above study also found that encapsulation of Gem, 5-fluorouracil, and oxaliplatin within Cet-PICs could further improve their efficacy in heterotypic organoids; the use of the nanoconstructs at a 1 μm lysophosphocholine BPD conjugate (BPD-PC) equivalent showed that Cet-PICs mediated a fractional viability of ~0.64 vs. 0.4 with chemotherapy-loaded Cet-PINs (*p* < 0.0001). Therefore, the delivery of high-payload, combination therapies is another inherent advantage of the nanoscale platform, which offers the opportunity of increasing the cytotoxic effect of PDT even further.

Though the greatest use of NPs has been to increase the cytotoxic effect on PDAC cells, efforts have also been underway to use NPs for stromal targeting, which would ultimately overcome the physical barrier it provides and improve treatment efficacy. Studies of NPs have demonstrated their ability to disrupt the crosstalk between stromal elements in PDAC. Saha et al. (2016) demonstrated that gold nanoparticles (AuNPs) alone could inhibit the proliferation and migration of PSCs and PDAC cells in vitro; a maximum of ~70–80% inhibition of proliferation at 48 h with 50 μg/mL AuNPs was seen in two PSC cell lines (CAF19 and iTAF) and two PDAC cell lines (AsPC1 and Panc-1) [188]. AuNPs could also alter ECM synthesis; immunoblot analysis showed a dose-dependent reduction in fibronectin, collagens I and III, and aSMA in PSCs. Importantly, the study indicated AuNP-mediated disruption of crosstalk between PSCs and PDAC cells. When treated with conditioned media (CM) of AuNP-treated PSCs, PDAC cell proliferation was decreased compared to the CM of the control PSCs without AuNP treatment. Subsequently, AuNP-mediated reduction in PSC activation was found to inhibit matrix deposition, enhance angiogenesis, and inhibit tumour growth in an orthotopic co-implantation model. Overall, the NPs seem to reprogram the TME through the targeting of stromal components, which reduces their reciprocal role in the development of the stroma and cancer itself. It is theorised that AuNPs have an inherent cytotoxic capability via their binding to biological molecules, particularly proteins, via cysteine and lysine residues. Though this mediates cell death through damage to the structure and biological function of these target molecules, the cytotoxic effect can be potentiated by the encapsulation of a PS molecule and the subsequent application of PDT.

A 2022 study found that the encapsulation of the PS protoporphyrin IX in a novel NP targeted using folic acid could also inhibit the crosstalk between the stroma and PDAC cells [123]. The NPs were able to mediate a dose-dependent decrease in cell viability following irradiation in both PSCs and Panc-1 cells and could interfere with the supportive effect of PSCs on Panc-1 cells; when co-cultivated, the apoptosis percentage of Panc-1 cells was 50% following NP-PDT, and this was the same when they were cultured alone. However, in a Gem-only-treated group, the apoptosis of Panc-1 cells was 31% in co-cultured cells and 50% when the cells were cultured alone. Therefore, though PSCs seem to provide a protective effect on Panc-1 cells to Gem, this can be overcome by NP-PDT. Furthermore, in the co-cultivation system, the proliferation of the Panc-1 cells was accelerated with Gem or no treatment, whereas the curve was unaffected following NP-PDT. Therefore, NP-PDT can interfere with the crosstalk between PDAC cells and the stroma. NP-PDT also had several important effects on PSCs. Quantitative polymerase chain reaction (qPCR) and Western blot analyses showed that NP-PDT could downregulate a-SMA and TGF-β and, in turn, reduce the expression of Col1 and fibronectin. Subsequent in vivo investigation showed that NP-PDT resulted in over 87% tumour volume reduction even in tumours with a large desmoplastic (PSC) component. Although the study did not compare the data to the use a ‘free PS’, which makes it difficult to determine the benefits of NP use, the data suggest that NP-mediated PDT can interrupt the crosstalk between stromal and PDAC cells, which increases the efficacy of the treatment and the subsequent chemotherapy intervention.

Another way of physically disrupting the PDAC stroma is to functionalise NPs with enzymes that can mediate its degradation. A recent 2022 study investigated the role of Dox-loaded AuNPs for the collagenase-mediated enzymatic degradation of the PDAC ECM in vivo [124]. The NPs effectively degraded ECM collagen, which facilitated their subsequent diffusion from the blood vessels to the distal tumour regions; the collagenase-functionalised NPs penetrated 3.31 times deeper than the control NPs without collagenase (*p* < 0.0001) and exhibited the best antitumour effect (tumour volume of ~50 vs. ~360 mm^3^ in controls 21 days post-treatment (*p* < 0.01). A similar study of Verteporfin-mediated PDT also showed that PSD enhanced the delivery of NPs to the tumour site in PDAC [189]. The authors attributed the better penetration of particles to the light-mediated destruction of ECM components contained within the Matrigel, including collagen and laminin. In Panc-1 spheroids co-cultured with fibroblasts, PDT improved therapeutic NP delivery; penetration of medium size NPs (0.1 or 0.2 μm) was visibly greater in spheroids with depleted stroma vs. the untreated cultures. The data suggest that PSD holds potential for improving the delivery of subsequent therapeutics by degrading the dense stroma. This effect could be further enhanced by targeting the PS directly to the stromal constituents in PDAC. For example, PS-containing NPs could be functionalised with molecules targeted specifically to PSCs, which would enhance the proposed effects of PSD, including collagen deposition or ECM crosslinking, as shown in Figure 5.

Recently, there have been developments toward several types of synthetic organic NPs for PDT application. These materials can benefit the use of NPs for PS delivery in PDT as they offer a strong absorbance at a long wavelength, a lower dark toxicity, and better metabolic features, among other benefits [190]. Two main designs have been considered: highly tailorable conjugated small molecules, which usually comprise a targeting ligand, linker, and drug payload, or polymers, larger structures prepared by crosslinked organic molecules via covalent bonds. One study developed a small molecular semiconducting perylene diimide (SPDI) for advanced phototheranostics; its amphiphilic properties allow the molecule to self-assemble into a stable yet soluble nanomicellar structure capable of carrying a PS [191]. Promising results have been demonstrated with SPDI molecules, which have shown favourable photothermal properties and an ability to inhibit tumour growth in vivo [192]. Of particular relevance to PDT, the SDPI molecules can be modified using polyethylene glycol (mPEG), which enhances their retention at the tumour site. Other peptide modifications might include the targeting of the structure directly to stromal components in PDAC to facilitate better PSD for PDT. In a similar study, the role of a semiconducting polymer nanostructure was investigated for combined radio- and photothermal therapy of PDAC [193]. The NPs demonstrated high CFPAC-1 cell uptake, excellent stability, and significant retention at the tumour site. This is a good indication for any future application of such nanostructures to PDT, which can potentiate the cytotoxic effect of the attached PS by targeting it specifically to the tumour or stromal components.

## 8. Conclusions

Although PDT for stromal depletion in PDAC is promising, there are several important aspects of its use yet to be clarified. Firstly, although preclinical studies have thus far not suggested that destroying the stromal components could contribute to tumour progression, we do not know that the same would be seen in practice. For example, the removal of the stroma may provide any residual cancer cells with a more aggressive phenotype due to its original tumour-suppressive functions. However, as the subsequent delivery of CT would be improved, the removal of residual cells may be more effective. Another consideration is how it might be possible to mitigate the effect of the desmoplastic stroma on the efficacy of PDT. Of particular concern is the hypoxic environment, which limits the efficacy of PDT treatment. However, efforts are underway to address this, including the use of vascular pruning and the more recent facilitation of in situ oxygen production or the delivery of oxygen microbubbles [167,168]. This emphasises that PDT for stromal depletion would need to be enhanced using various strategies in order to see a maximum benefit. One other consideration is the suitability of PDT-mediated stromal depletion for different disease stages. So far, clinical trials have focused on its use for locally advanced PDAC as only modest improvements in survival have been observed in this setting using other therapies. However, its use may also be beneficial for BR PDAC to improve the efficacy of NACT and increase the chances of achieving a resectable tumour state. Similarly, it could also be adopted in the metastatic setting to improve the outcomes of palliative CT, though further investigation would be needed to address this, particularly as the composition of the stroma may also vary with the disease stage.

The current preclinical evidence points towards the suitability of PDT for stromal depletion in PDAC. In addition to its cytotoxic effect on the disease itself, the use of low-dose light delivery or ‘photodynamic priming’, which induces a lower level of intracellular ROS, as determined by the regimen choice or depth of light penetration [194], could help destroy the desmoplastic stroma and resensitise cells to subsequent therapies. This would enable lower doses of chemotherapeutics to be given, which in turn would decrease toxic side effects, help avoid the selection pressures imposed by high doses, and increase the chances of successful surgical resection.

## Data Availability

Data sharing not applicable. No new data were created or analysed in this study. Data sharing is not applicable to this article.
